# Implementing preexposure prophylaxis among key populations: an opportunity for patient-centered services and management of hepatitis B

**DOI:** 10.1097/QAD.0000000000001749

**Published:** 2018-03-21

**Authors:** Joseph Larmarange, Valentine Becquet, Jean-Marie Masumbuko, Marcellin Nouaman, Mélanie Plazy, Christine Danel, Serge Eholié

**Affiliations:** a Centre Population et Développement, Institut de Recherche pour le Développement, Université Paris Descartes, Inserm, Paris, France; b Programme PAC-CI, Abidjan, Côte d’Ivoire; c Bordeaux Population Health Research Center UMR 1219, ISPED, Université de Bordeaux, Inserm, Bordeaux, France.

When taken properly, Tenofovir-based oral preexposure prophylaxis (PrEP) has been proven to be efficient to prevent HIV acquisition [1–5]. Since 2015, PrEP is recommended by the WHO for populations at ‘substantial risk’ of HIV [6]. However, WHO points out the need for additional research on PrEP in ‘real life’ on questions such as demand creation for oral PrEP; best delivery models in different contexts and for different populations; social and behavioral impact of PrEP; or integration of PrEP services with other services [6]. Transitioning from efficacy trials to implementation requires to adapt interventions. Preliminary research (ANRS 12361 PrEP-CI [7]) has been conducted in Côte d’Ivoire (CI) in collaboration with community non-governmental organizations to explore relevance and feasibility of implementing a PrEP program among female sex workers, one of the most exposed populations countrywide (estimated HIV prevalence: 29% [8]). The following observations emerged from that collective work.

All efficacy PrEP trials provided a range of sexual healthcare services in addition to PrEP drugs [9–11]. Such services appeared essential for any PrEP program. By design, they were conditional to PrEP use. However, regardless of their interest in using PrEP, female sex workers interviewed in Côte d’Ivoire, and more broadly key populations worldwide [12–16], have many unmet sexual and reproductive health needs: sexually transmitted infections screening and care, contraception and birth control, menstrual management, addictions and risky behaviors… When transitioning to real life, we should not reproduce the service model of efficacy PrEP trials, that is a PrEP program with additional services. Instead, a paradigm shift toward a patient-centered approach should be preferred, that is offering sexual and reproductive health services in which PrEP is an option but not mandatory.

In Western and Central Africa, the prevalence of hepatitis B is relatively high [17]. In Cote d’Ivoire, more than 11% of new blood donors were positive for hepatitis B surface antigen in 2008–2012 [18]. Tenofovir is also used for hepatitis B treatment. But, currently, treatment is not free for monoinfected hepatitis B patients, whereas it is covered by AIDS programs for HIV-hepatitis B coinfected patients. In such context, it would be ethically unacceptable to provide free HIV PrEP without taking into account patients in needs of hepatitis B treatment. Actually, for those patients, offering Tenofovir-based HIV PrEP constitutes an opportunity to simultaneously treat their hepatitis B. It requires to integrate WHO recommendations on hepatitis B [19] within PrEP guidelines [20], possibly to simplify hepatitis B care algorithms and to allow hepatitis B care in decentralized sexual health clinics and not only in hospital services. Most efficacy PrEP trials excluded hepatitis B patients. Additional clinical research exploring interactions between HIV PrEP and hepatitis B treatment, in particular the risk of flare if PrEP is stopped, is required.

PrEP programs could be built on the existing community services for HIV care and treatment. Providing services for HIV positives and HIV negatives within the same clinics could be a way of minimizing the stigma associated with entry and retention into HIV care. In addition, HIV patients have unmet sexual and reproductive health needs as well. Integrating services together and transforming HIV clinics into sexual health clinics could lead to many health outcomes improvements and also to possible cost sharing and savings.

So far, the focus of HIV programs has mainly been on reaching individuals never tested for HIV, identifying new positives and linking them to HIV care and treatment. Transitioning PrEP from trials to implementation constitutes an opportunity for developing people-centered approaches integrating all sexual and reproductive health services together, including hepatitis B. It is crucial to avoid a silo-based perspective in which services are separated from each other. Moving from HIV care clinics to sexual health clinics would allow to globally improve the health of key populations and their partners, beyond HIV outcomes alone. To ensure the success of new prevention programs, we have to take the next step forward. Beyond biomedical innovations, innovations in terms of intervention implementation, delivery models and public health policies are urgently required [21], in particular in Western and Central Africa [22]. Scaling-up PrEP is a key moment. We should not miss out on this opportunity.

## Acknowledgements

The PrEP-CI ANRS 12361 was funded by the Bill and Melinda Gates Foundation and the French National Agency for AIDS and Viral Hepatitis Research (ANRS).

Author contributions: J.L. and V.B. wrote the article. All authors contributed to the interpretation, reviewed the article and approved the final version of the article.

### Conflicts of interest

There are no conflicts of interest.
